# Novel echocardiographic tools for predicting a good response to cardiac resynchronization therapy: a narrative review

**DOI:** 10.3389/fcvm.2026.1906344

**Published:** 2026-08-25

**Authors:** Beata Uziębło-Życzkowska, Despina-Manuela Toader, Magdalena Potapowicz-Krysztofiak

**Affiliations:** 1 Department of Cardiology and Internal Diseases, Military Institute of Medicine – National Research Institute, Warsaw, Poland; 2 EuroEchoLab, Emergency County Clinical Hospital Craiova, Craiova, Romania

**Keywords:** cardiac resynchronization therapy, longitudinal strain, mechanical dyssynchrony, myocardial work, speckle tracking echocardiography

## Abstract

Despite significant progress in new echocardiographic techniques, the role of echocardiography in qualifying patients for cardiac resynchronisation therapy (CRT) remains limited solely to the assessment of left ventricular ejection fraction (LVEF). This review aims to present the current knowledge on new echocardiographic methods that may be useful in predicting the response to resynchronization. Recent advances in speckle-tracking echocardiography (STE) have introduced several promising parameters. Measures such as global longitudinal strain, mechanical dispersion, and various indices of mechanical dyssynchrony, including those derived from myocardial work, have shown growing evidence of their utility in identifying patients more likely to benefit from CRT. Increasing data support the use of these deformation-based techniques as complementary tools to traditional LVEF assessment, offering a more precise characterization of left ventricular mechanics and potentially improving patient selection. This paper aims to demonstrate how new echocardiographic techniques allow for the evaluation of relevant left ventricular mechanical dyssynchrony, and to highlight their current and emerging value in the clinical assessment and management of heart failure patients being considered for CRT.

## Introduction

Despite significant progress in new echocardiographic techniques, the role of echocardiography in qualifying patients for cardiac resynchronisation therapy (CRT) is limited solely to the assessment of left ventricular ejection fraction (LVEF). The unfavorable results of the PROSPECT study (1), followed by the Echo CRT trial (2), did not improve this situation. Indications for CRT are still primarily based on the evaluation of QRS width and morphology on electrocardiography (ECG), as well as LVEF on echocardiography. In symptomatic heart failure (HF) patients in sinus rhythm (SR) with an LVEF ≤ 35% despite optimal medical therapy, CRT is recommended when QRS duration is ≥150 ms and the QRS morphology is typical left bundle branch block (LBBB), and it should also be considered when QRS duration is 130–149 ms with LBBB morphology, or ≥150 ms with non-LBBB morphology (3).

Since the introduction of CRT in 2000, referred to as the “device era” (4), it has become a breakthrough therapy for patients with heart failure with reduced ejection fraction (HFrEF) and wide QRS complexes. At the same time, it has triggered a surge of research evaluating the effectiveness of the therapy and the predictive factors of its success.

In recent years, the progressive development of advanced echocardiographic techniques have significantly increased both scientific interest and clinical expectations regarding their potential to improve patient selection for CRT. Novel imaging modalities, such as speckle-tracking echocardiography (STE), have provided deeper insights into cardiac mechanics and dyssynchrony assessment. These advancements offer the possibility of identifying patients who are most likely to benefit from CRT more accurately, thereby enhancing therapeutic efficacy and reducing the proportion of non-responders. Consequently, echocardiographic evaluation has become an increasingly central element in refining CRT indication and optimizing clinical outcomes.

This paper aims to illustrate how novel echocardiographic techniques enable the assessment of significant LV mechanical dyssynchrony, and subsequently to present their current and promising role in the clinical evaluation and management of patients with HF who are candidates for CRT.

## Methods

This article is a narrative (non-systematic) review. We searched PubMed/MEDLINE, Scopus and the Cochrane Library for English-language articles published up to April 2026, using combinations of terms related to cardiac resynchronisation therapy and its response, left bundle branch block and conduction system pacing, and to speckle-tracking echocardiography-derived parameters of left ventricular deformation, mechanical dyssynchrony and myocardial work. The reference lists of retrieved articles and of relevant guidelines were screened manually to identify additional sources. We prioritised randomised controlled trials, prospective and retrospective cohort studies, systematic reviews and meta-analyses, current European Society of Cardiology guidelines and consensus documents that reported echocardiographic predictors of CRT response or evaluated deformation-based and myocardial work parameters. Studies were selected on the basis of their relevance to the prediction of CRT response and to the methodological assessment of LV mechanical dyssynchrony. Case reports, conference abstracts without an available full text, and non-English publications were generally excluded. As this is a narrative review, no formal quantitative synthesis or risk-of-bias scoring was performed.

### Inconsistent definition of CRT response

The first challenge in these studies is the lack of fully uniform criteria for CRT effectiveness, the definition of so-called CRT responders. Across major trials, response criteria vary considerably, complicating comparisons of outcomes. The most commonly used echocardiographic marker is reverse left ventricular (LV) remodeling, typically defined as ≥10%–15% reduction in LV end-systolic volume (LVESV) or ≥5%–10% increase in LVEF after 6–12 months of therapy. Some studies rely primarily on clinical criteria such as NYHA improvement, reduced HF hospitalizations, or composite endpoints. Despite the consistent association of reverse remodeling with better prognosis, the lack of standardized response criteria remains a major limitation in CRT research and clinical practice. Across studies in this topic, various parameters continue to be used in its assessment (5, 6).

The magnitude of such reverse LV remodeling has been shown to strongly predict long-term survival benefits and a lower incidence of heart failure hospitalizations (7, 8). Therefore, structural response assessed by echocardiography remains one of the most reliable indicators of prognostic benefit after CRT, although non-response continues to affect approximately 30%–40% of treated patients (3). Table 1 presents the main echocardiographic parameters used in previous studies to assess response to CRT.

**Table 1:** Main echocardiographic response criteria used in previous CRT studies.

No.	Study	Type of response criterion/specific threshold	Time of assessment
1	PROSPECT (2008) (1)	Echocardiographic (LV reverse remodeling) + clinical composite. Super-responders: reduction in LVESV ≥ 30%; responders: 15%–29% reduction; also combined clinical + echo: reduction ≥15% + clinical improvement.	6 months
2	REVERSE (2015) (7)	Echocardiographic (LV remodeling). LVESVi reduction ≥ 15%.	∼12 months
3	MADIT-CRT (2013) (8)	Echocardiographic (LV volumes) + clinical outcome. Percent change in LVEDV; also LVESV reductions linked to outcome.	12 months
4	CARE-HF (2009) (9)	Echocardiographic (LV volumes/function) + clinical outcomes. Sustained reduction in LV volumes/increase LVEF; no single fixed threshold.	3–18 months, up to ∼29 months
5	SMART-CRT (2024) (10)	Echocardiographic (LV reverse remodeling). Defined response as >15% reduction in LVESV at 6 months.	6 months
6	SMART-AV (design/pooling) (11)	Echocardiographic (LV reverse remodeling). CRT response defined as ≥15% reduction in LVESV at ∼6 months.	∼6 months
7	MORE-CRT MPP (12)	Echocardiographic (LV remodeling). Non-responders defined as <15% reduction in LVESV; responders ≥15% reduction.	∼6 months

CRT, cardiac resynchronisation therapy; LV, left ventricle; LVEDV, left ventricular end-diastolic volume; LVEF, left ventricular ejection fraction; LVESV, left ventricular end-systolic volume; LVESVi, left ventricular end-systolic volume indexed to BSA; BSA, body surface area.

### Speckle tracking echocardiography in the assessment of mechanical dyssynchrony of the left ventricle

Speckle-tracking echocardiography, a relatively new and increasingly used echocardiographic technique, allows for the assessment of LV mechanics and mechanical dyssynchrony. Therefore, it may provide valuable additional information that could improve the selection of patients eligible for CRT. In the systematic review and meta-analysis by Bazoukis et al. (13) twelve studies encompassing about 1,000 patients (mean age 63.8 years, approx. 69% male) undergoing CRT were evaluated. Patients who responded to CRT had significantly better baseline global longitudinal strain (GLS) values than non-responders [mean difference ∼−2.13% (−3.03, −1.23), *p* < 0.001]. Moreover, CRT responders also showed greater improvements in GLS during follow-up compared with non-responders [mean *Δ*GLS difference ∼−3.20% (−4.95, −1.45), *p* < 0.001]. The authors conclude that GLS, both at baseline and its change over time, may serve as an important echocardiographic tool to better select patients for CRT and to define responders. However, the role of STE in the evaluation of patients with HFrEF who are potential candidates for CRT is not limited to the assessment of GLS. New echocardiographic parameters derived from STE analysis described underneath provide additional information that enables more precise quantification of LV dyssynchrony.

It should be emphasised that the studies cited above assessed mainly GLS. However, the direction in which deformation is measured may itself affect the predictive value of dyssynchrony analysis. Speckle-tracking echocardiography can also measure radial and circumferential strain, and several studies suggest that radial strain may identify CRT responders better than longitudinal or circumferential strain. Delgado et al. (14) compared all three strain directions in 161 CRT candidates. Baseline dyssynchrony differed between responders and non-responders only for radial strain: a radial dyssynchrony cut-off of ≥130 ms predicted response with 83% sensitivity and 80% specificity, whereas longitudinal and circumferential strain were not discriminative. In the prospective multicentre Speckle Tracking and Resynchronization (STAR) study, Tanaka et al. (15) similarly found that dyssynchrony measured by radial and transverse strain was associated with both LVEF response and long-term event-free survival after CRT. In contrast, circumferential and longitudinal strain predicted response only when dyssynchrony was present, but failed to detect it in about one-third of patients who responded. These findings show that the direction of strain analysis matters, and that radial deformation may add to, and in some respects outperform, GLS alone in detecting mechanical dyssynchrony.

### Left ventricle strain patterns in typical left bundle branch block

Typical LBBB is a classic example of LV electromechanical dyssynchrony, which contributes to the development and progression of LV dysfunction and heart failure. The large meta-analysis by Martins et al. (16) demonstrated that the presence of LBBB morphology was the strongest independent predictor of echocardiographic response to CRT. Patients with LBBB showed significantly higher rates of LV reverse remodeling compared with those without LBBB, confirming its key role in appropriate patient selection for CRT. These findings highlight the importance of a detailed echocardiographic assessment of typical LBBB patterns in the context of decision-making and predicting the degree of response to CRT.

Strict ECG criteria for LBBB were published by Strauss et al. (17). However, only by understanding the relationships between the electrical and mechanical manifestations of LBBB can we gain better insight into LBBB physiology, which may lead to improved selection of CRT responders. LV strain analysis, as an advanced echocardiographic tool, allows the detection of mechanical delay and contraction heterogeneity of the LV operating under LBBB conditions.

In typical LBBB, two-dimensional speckle-tracking echocardiography (2D-STE) demonstrates a distinctive pattern of LV mechanical dyssynchrony. The septal wall initiates contraction early, often in the isovolumetric phase, while the lateral wall is concurrently passively stretched rather than actively contracting. Only afterwards does the lateral wall commence its own contraction, frequently reaching its maximal shortening after aortic valve closure. Risum et al. (18), defined echocardiographic criteria for so-called “true LBBB”, combining three criteria (1): early shortening of ≥1 basal or mid-ventricular segment in the septal wall accompanied by early stretching in ≥1 basal or mid-ventricular segment in the lateral wall; (2) early septal peak shortening occurring within the first 70% of the ejection phase; and (3) lateral wall peak shortening after aortic valve closure. They also demonstrated that concordance between ECG and echocardiographic criteria in identifying “true LBBB” most accurately predicts CRT response: thirty-six (95%) of the 38 patients with concordant LBBB classification responded to CRT (16). Duchenne et al. (19) demonstrated that LBBB-related septal strain patterns, categorized into five strain-based stages (LBBB-0 to LBBB-4), show a clear stepwise relationship with CRT outcomes. Higher LBBB stages were associated with significantly greater LV reverse remodeling and improved survival after CRT, whereas lower stages showed attenuated benefit. Myocardial scar burden correlated inversely with LBBB stage but did not independently predict outcomes after adjustment, while strain stage remained an independent predictor of both volumetric response and survival. Importantly, CRT-treated patients with higher LBBB stages experienced substantially greater survival benefit compared with conservatively treated CRT-eligible patients, supporting the value of strain-based staging as a robust framework for identifying patients most likely to respond to CRT.

These observations agree with earlier mechanistic work by Leenders et al. (20). Using speckle-tracking longitudinal septal strain together with the CircAdapt computer model in 132 CRT candidates with LBBB-type morphology, they identified characteristic septal deformation patterns, most often a double-peaked systolic shortening. These patterns arise specifically from intraventricular dyssynchrony, whereas abnormal regional contractility (e.g., scar) progressively changes and blunts them. The authors concluded that the septal deformation pattern reflects both dyssynchrony and regional contractility, and therefore predicts CRT response. This provides a physiological basis for the more recent strain-based LBBB staging systems, in which more pronounced, “typical” deformation patterns indicate a mainly dyssynchrony-driven, and therefore reversible, substrate.

**Figure 1:**
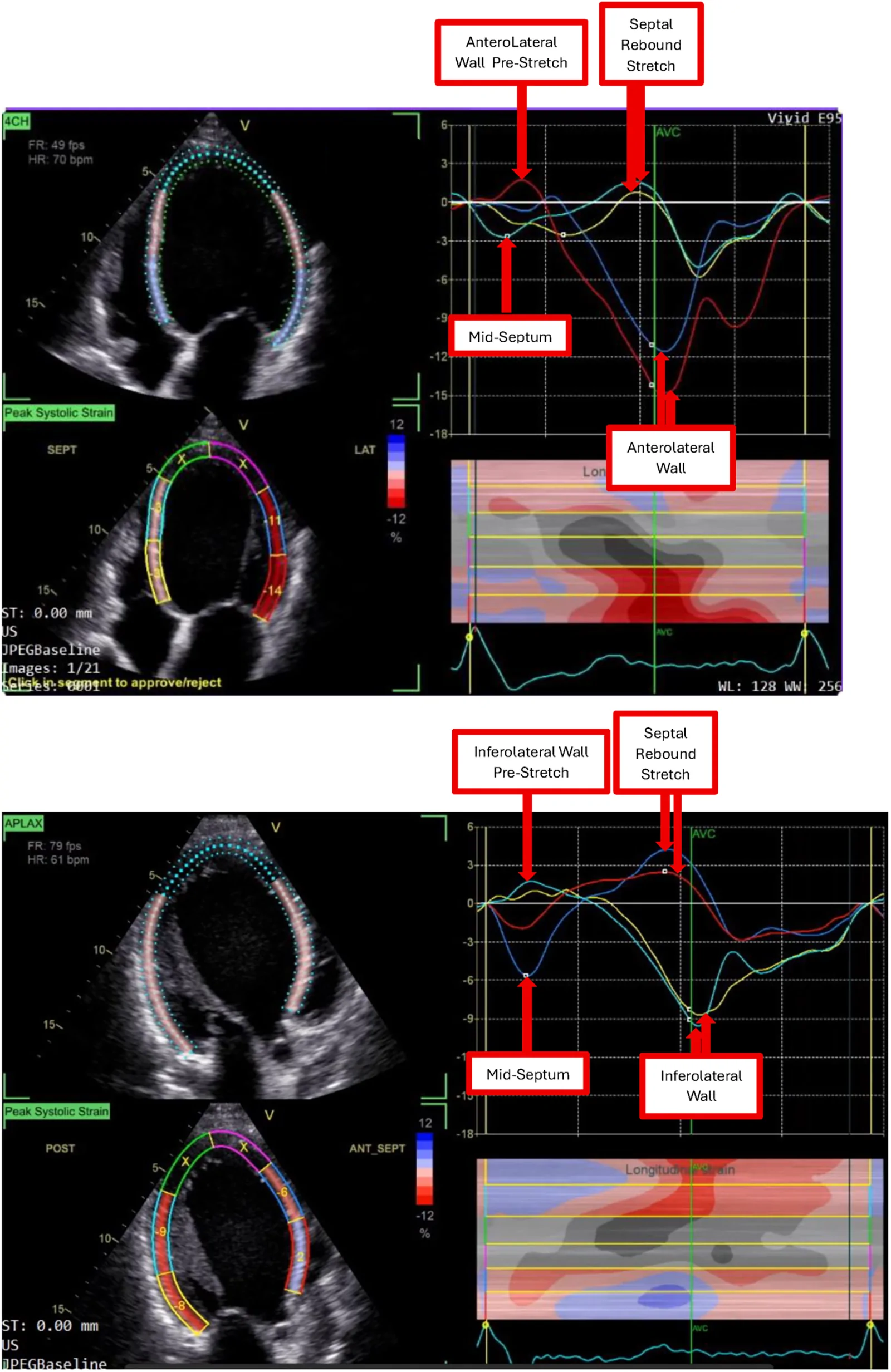
Typical LBBB contraction pattern visualized using 2D-STE in the apical four-chamber (upper panels) and apical long-axis/three-chamber (lower panels) views (for better clarity of the image, the curves for the apical segments were removed). Early peak strain of the septum is followed by continued stretching during the remainder of systole. Similar patterns are observed in 3AC. 2D-STE, two-dimensional speckle-tracking echocardiography; 3AC, apical long-axis (three-chamber) view; 4AC, apical four-chamber view; LBBB, left bundle branch block.

This abnormal pattern of LV contraction in LBBB, clearly visualized using 2D-STE, is presented in Figure 1.

Such regional differences in the function of individual LV segments lead to significant disparities in the mechanical work performed by each LV segment. The basal (and potentially mid-ventricular) segments of the lateral/posterior wall become overloaded, resulting in their gradual thickening, whereas the basal septal/anteroseptal segments undergo thinning, as demonstrated in the study by Cvijic et al. (21). Only the restoration of a proper LV contraction pattern following CRT implantation can interrupt this harmful vicious cycle and reverse the unfavourable LV remodeling. In the study by Calle et al. (22), “true LBBB”, LBBB-stage 4, was associated with significant LBBB-induced remodeling and predicted a favourable response, or even a super-response, after CRT. In this study, the other echocardiographic LBBB patterns (those different from “true LBBB”) showed less LBBB-induced LV remodeling, which was associated with less reverse remodeling after CRT. The authors suggest that the proposed LBBB classification may help identify which patients will benefit most from CRT, as advanced LBBB stages (“true LBBB”) indicate substantial LBBB-induced remodeling and predict stronger reverse remodeling after CRT. On the contrary, non-typical LBBB pattern implies that LBBB is not the predominant cause of LV dysfunction, and thus these patients are less likely to respond to CRT.

### Speckle tracking echocardiography in the assessment of LV mechanical dispersion

Mechanical dispersion (MD) measured by speckle-tracking longitudinal strain has emerged as a next promising predictor of response to CRT. By calculating the standard deviation of time to peak negative longitudinal strain across usually 17-segment models of the LV, this parameter quantifies the temporal heterogeneity of myocardial contraction. Recent research has strengthened the role of STE–derived mechanical dyssynchrony parameters in identifying CRT responders. In the LEVEL-AT trial secondary analysis (23), conduction system pacing led to significant improvements in mechanical dyssynchrony and global strain over time, demonstrating that dyssynchrony correction is closely linked to favourable LV functional response. Similarly, Zhu et al. (24) showed that baseline mechanical dispersion and GLS were independent predictors of response to multipoint pacing, with higher mechanical dispersion identifying patients more likely to benefit from CRT-based resynchronisation.

Together, these studies support the clinical utility of mechanical dyssynchrony assessment, particularly mechanical dispersion, as a complementary tool to refine patient selection and improve prediction of CRT outcomes beyond conventional ECG criteria alone.

Hara et al. (25) demonstrated that the assessment of mechanical dyssynchrony is particularly important in patients without typical LBBB. In this group, the presence of mechanical dyssynchrony identified individuals who achieved long-term outcomes comparable to LBBB responders, whereas non-LBBB patients without dyssynchrony had the poorest prognosis. These findings support the clinical value of mechanical dyssynchrony assessment, such as mechanical dispersion, to refine CRT candidate selection beyond QRS morphology alone.

In clinical practice, MD may complement conventional electrophysiological criteria and visual markers such as septal flash or apical rocking, especially in patients with intermediate QRS or non-classic conduction patterns. Prospective validation in large multicentre cohorts remains necessary before widespread adoption. However, MD adds mechanistic insight into mechanical dyssynchrony and may refine patient selection for CRT.

**Figure 2:**
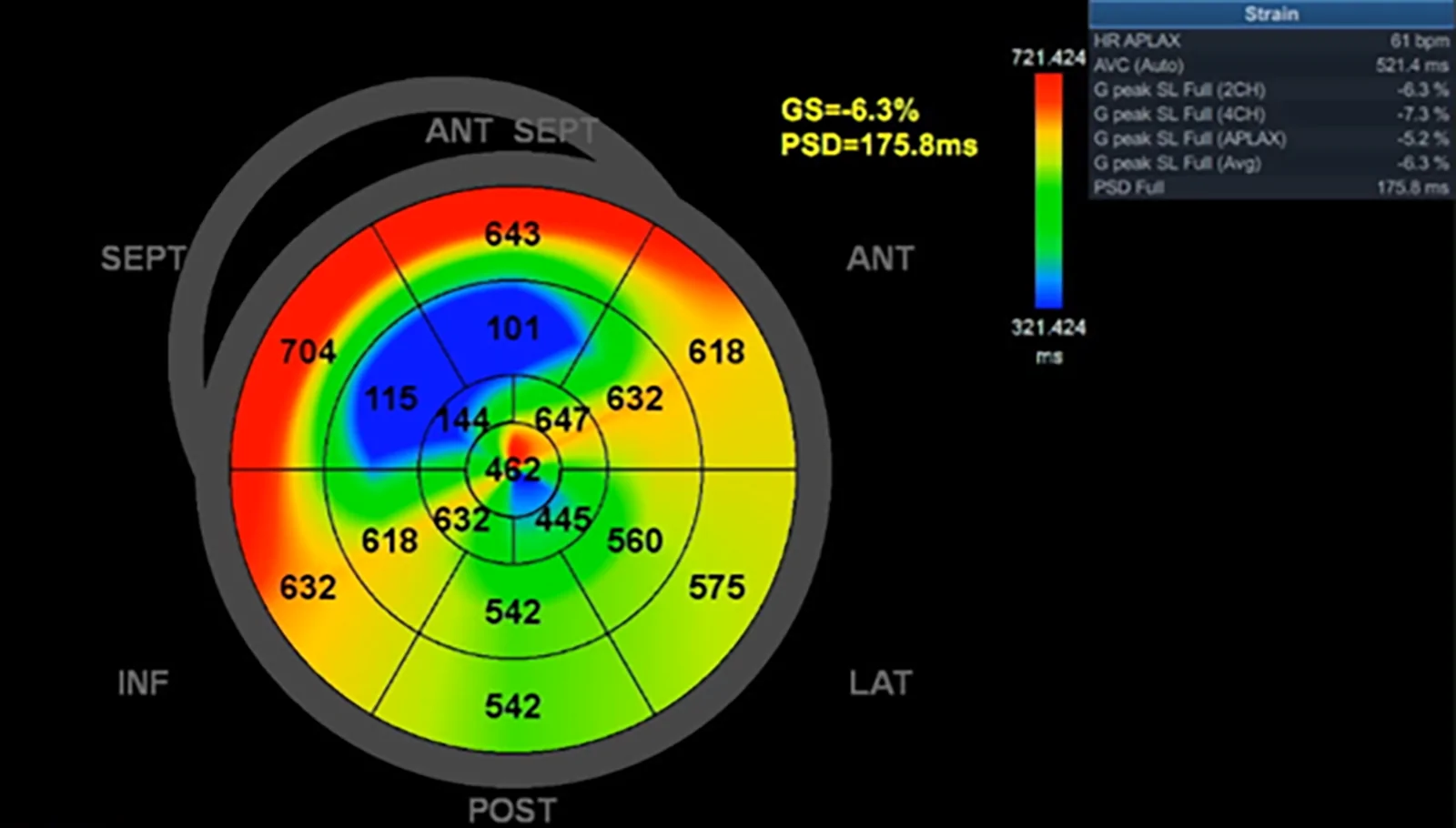
Left ventricular mechanical dispersion in a patient with typical LBBB measured by speckle-tracking echocardiography. The bull's-eye (polar) plot illustrates the timing of peak longitudinal strain in each LV segment. In this patient with LBBB, the early activation of the septal segments (blue areas, shorter time to peak strain) contrasts with the markedly delayed contraction of the lateral wall (yellow zones, longer time to peak strain). This electromechanical dyssynchrony leads to increased mechanical dispersion, quantified as the standard deviation of time-to-peak strain across all segments (PSD = 175.8 ms). The global longitudinal strain (GLS) is reduced (−6.3%), consistent with impaired systolic function and asynchronous contraction typical for LBBB. GLS, global longitudinal strain; LBBB, left bundle branch block; LV, left ventricle; PSD, peak-strain dispersion (standard deviation of the time to peak longitudinal strain across all LV segments).

Figure 2 shows an example of left ventricular mechanical dispersion measured by speckle-tracking echocardiography in a patient with typical LBBB.

### Left ventricular rotation and torsion in predicting CRT response

Left-ventricular rotation and torsion represent key components of myocardial mechanics, reflecting the coordinated wringing motion generated by subendocardial and subepicardial fiber orientation. In patients undergoing CRT, these rotational parameters have been proposed as potential markers of mechanical dyssynchrony and predictors of therapeutic response.

Several studies have shown that impaired LV twist and torsion are frequently present in non-responders to CRT and may improve immediately after initiation of biventricular pacing. Early increases in peak LV twist have been associated with favourable reverse remodeling and higher likelihood of clinical improvement. In particular, studies using speckle-tracking and three-dimensional echocardiography demonstrated that responders typically exhibit an acute increase in apical rotation and global torsion during CRT, whereas non-responders may show little change or even a reduction in torsional mechanics.

Bertini et al. (26) examined 80 HF patients treated with CRT and found that LV twist mechanics are closely linked to treatment response. At baseline, CRT candidates had markedly reduced LV twist compared with healthy controls. During follow-up, only responders demonstrated a significant increase in LV twist along with improvements in LVEF and LV volumes, while non-responders showed no recovery of torsional function. The strongest predictor of CRT response was the immediate rise in LV twist after CRT activation, indicating effective mechanical resynchronisation. Improvement in twist was also dependent on LV lead placement, with apical or mid-ventricular positions yielding the best results.

In a cohort of 48 HF patients undergoing CRT, responders showed a clear acute increase in global and apical LV torsion during CRT-on, whereas non-responders demonstrated a reduction in torsional values (27). Improvement in global torsion (>0.035°/cm) predicted 6-month reverse remodeling with good accuracy [area under curve (AUC) 0.80; sensitivity 88%; specificity 68%]. Concordance between the region of latest mechanical activation and LV lead position was associated with greater torsional improvement. Overall, the study demonstrated that early enhancement of LV torsion during CRT is strongly linked to subsequent reverse remodeling and may serve as an early predictor of CRT response.

The recent study evaluated 46 HF patients undergoing CRT and examined changes in LV rotational mechanics using STE (28). At 6 months, 48% were CRT responders. Responders showed a clear improvement in LV twist and in the novel twist integral (TI) immediately after CRT optimization, whereas non-responders showed little or no improvement. The increase in effective stroke volume was the strongest independent predictor of CRT response, followed by changes in TI and peak systolic twist. TI demonstrated good discriminatory value (AUC 0.86) for identifying responders. Overall, the study shows that early enhancement of LV rotational mechanics is closely linked to positive CRT remodeling.

Despite these promising findings, the clinical utility of LV rotational parameters remains limited by methodological variability, small sample sizes, and dependence on high-quality echocardiographic imaging. Differences in acquisition protocols and definitions of torsion also hinder standardization across centres. Consequently, although LV rotation and torsion offer mechanistic insight into electromechanical coordination and may help identify patients with favourable mechanical reserve, they are not yet recommended as routine predictors of CRT response.

**Figure 3:**
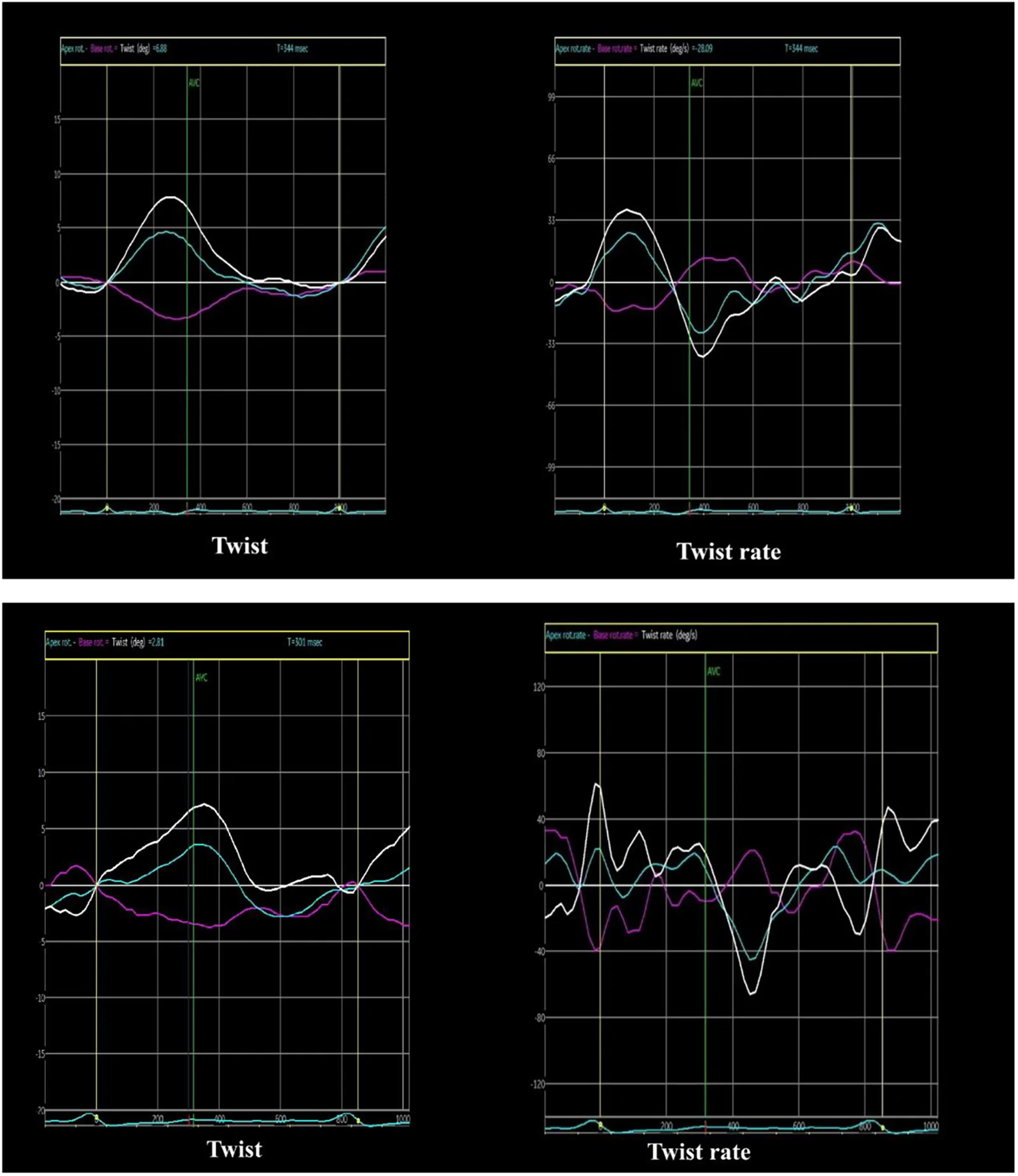
Torsional mechanics evaluated by 2D STE in normal condition (above) and in a patient with dilated cardiomyopathy and LBBB (below). In the normal heart (upper panels), LV twist increases smoothly and progressively during systole to a single, well-defined positive peak around aortic valve closure (AVC), and the twist-rate curve shows an orderly systolic peak followed by a coordinated diastolic untwisting (recoil). In the patient with dilated cardiomyopathy and LBBB (lower panels), peak systolic twist is markedly reduced and temporally delayed, and both the twist and twist-rate curves become irregular and multiphasic with loss of the coordinated systolic peak, reflecting impaired and dyssynchronous rotational mechanics. 2D STE, two-dimensional speckle-tracking echocardiography; AVC, aortic valve closure; LBBB, left bundle branch block; LV, left ventricle.

Further research with larger cohorts, harmonized measurement protocols, and standardized reference values is required to clarify the role of LV torsional mechanics in patient selection and optimization of CRT. Figure 3 presents torsional mechanics assessed by 2D STE in a normal heart and in a patient with dilated cardiomyopathy and LBBB.

### The role of myocardial work indices

Assessment of GLS in STE can identify subclinical LV dysfunction and LV dyssynchrony. However, similarly to LVEF, it is influenced by load dependency, which means that GLS values are susceptible to changes in both preload and afterload (29). In 2012, Russell et al. (30) introduced a new method for noninvasively assessing myocardial work (MW) and myocardial metabolism. In their study, the authors presented a non-invasive pressure–strain analysis technique that enables quantification of regional myocardial work. The method combines non-invasively estimated LV pressure curves with speckle-tracking–derived strain to calculate regional work (31). They demonstrated a robust correlation between noninvasively assessed regional myocardial work and invasively obtained pressure–strain loops, as well as regional glucose metabolism assessed by positron emission tomography. MW provides a more load-independent measure of LV performance than GLS because it incorporates afterload estimation using cuff blood pressure. This approach may support more accurate selection of candidates for CRT and help optimize device settings, potentially improving response rates.

Myocardial work analysis performed by transthoracic echocardiography provides four key parameters:

1. **Global Constructive Work (GCW)**—the amount of “constructive” work performed by the LV, defined as myocardial shortening during periods of increasing pressure and myocardial lengthening during periods of decreasing pressure. It reflects the effective systolic performance of the LV.
2. **Global Wasted Work (GWW)**—the amount of “wasted” work, represented by myocardial lengthening during pressure rise and myocardial shortening during pressure fall. It indicates mechanical inefficiency and is typically increased in the presence of dyssynchrony or regional dysfunction.
3. **Global Work Index (GWI)**—the total work performed by the LV from isovolumetric contraction to isovolumetric relaxation, expressed as the area within the pressure–strain loop. It represents an integrated measure of LV loading and systolic function.
4. **Global Work Efficiency (GWE)**—the ratio of constructive work to the sum of constructive and wasted work [GCW/(GCW + GWW)]. It reflects the proportion of total myocardial work that is effectively translated into pump function.

### Myocardial work in left bundle branch block

Left bundle branch block profoundly alters the pattern of LV electrical activation, producing mechanical dyssynchrony and characteristic changes in MW indices. Because MW integrates pressure–strain loops with non-invasive LV pressure estimates, it reflects not only deformation but also its energetic efficiency, making it particularly sensitive to the abnormalities introduced by LBBB.

In LBBB, GWI and GWE are typically reduced (32). The delay in septal activation causes early, ineffective septal contraction followed by excessive workload of the lateral wall. Despite often preserved or only mildly reduced LVEF, the overall work performed during systole falls because part of septal deformation occurs before effective LV pressure rise, lowering constructive work. GCW, the component of work contributing to effective LV ejection, is also decreased in LBBB. Much of the septal shortening is “wasted” during the pre-ejection period, and lateral wall contraction is temporally shifted. This leads to diminished synchrony of segmental work and reduces the net amount of global constructive systolic work. In contrast, GWW is markedly increased. Mechanically, the septum often shows early systolic stretch during lateral wall contraction and late shortening when lateral wall fibers are already relaxing. This paradoxical strain pattern results in a significant rise in wasted work, energy consumption that does not contribute to stroke volume. As a consequence of reduced GCW and increased GWW, GWE is consistently impaired in LBBB. Typical values fall below those seen in patients with normal conduction, often dropping into a range suggestive of mechanical dyssynchrony and potential responsiveness to CRT, especially when combined with reduced LVEF.

**Figure 4:**
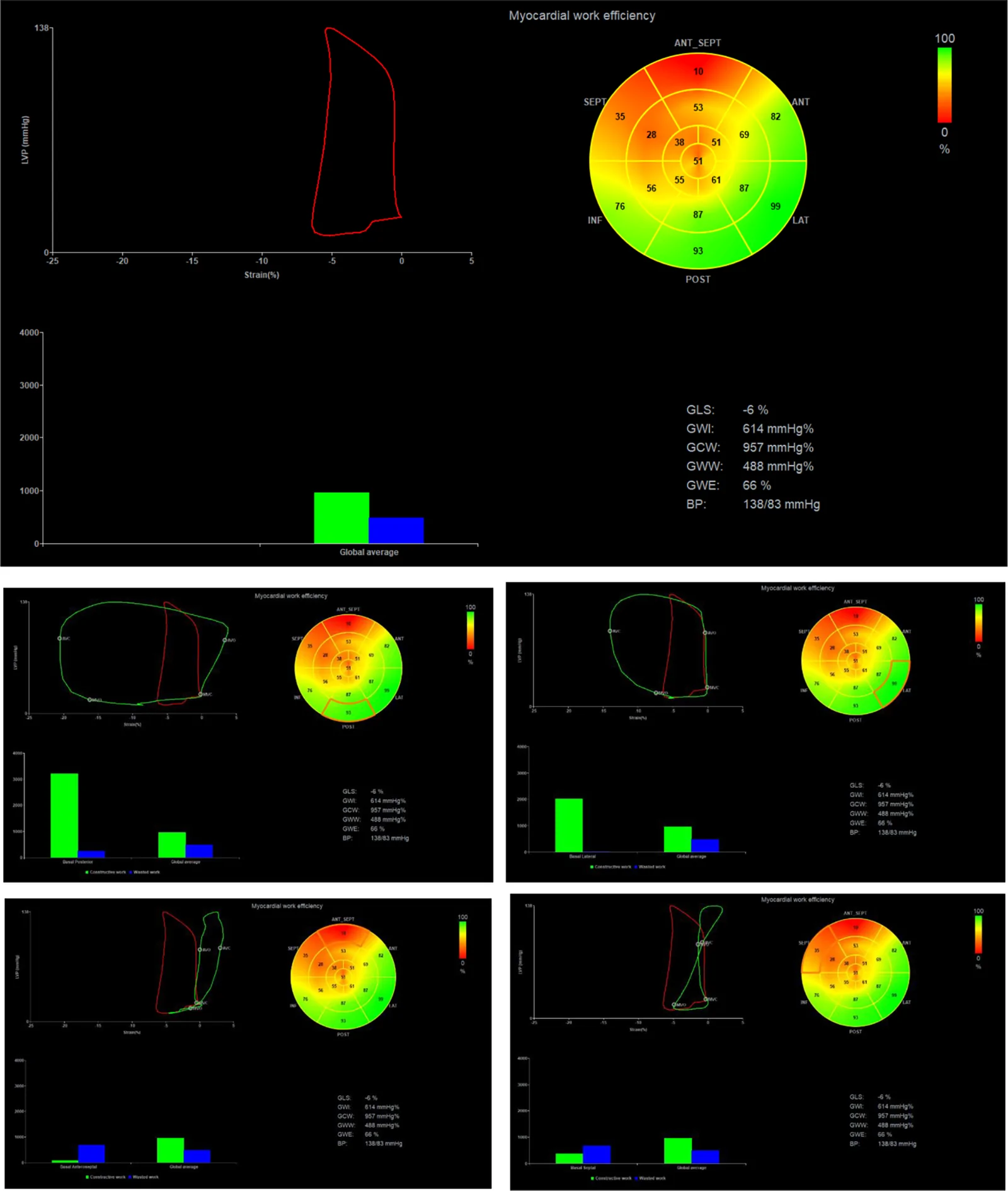
Global and regional myocardial work (MW) abnormalities in left bundle branch block (LBBB). The first panel presents global myocardial work parameters, including global longitudinal strain (GLS), global work index (GWI), global constructive work (GCW), global wasted work (GWW), and global work efficiency (GWE), together with the corresponding non-invasive pressure–strain loop and the average constructive vs. wasted work distribution. These values reflect overall mechanical performance of the left ventricle. The subsequent four panels illustrate regional myocardial work patterns characteristic of LBBB. In each regional assessment, the pressure–strain loop demonstrates typical mechanical dyssynchrony, with early paradoxical shortening of the septum and delayed, dominant contraction of the lateral wall. The bull's-eye maps show a consistent pattern of reduced constructive work and low work efficiency in the septal and anteroseptal segments, accompanied by compensatory increases in constructive work within the lateral and posterior walls. Bar graphs further highlight these regional disparities, with markedly elevated wasted work in septal regions and predominance of constructive work in the lateral myocardium. Together, these visualizations depict the classic redistribution of myocardial work in LBBB, septal work wasting, delayed lateral activation, regional mechanical imbalance, and globally reduced efficiency despite preserved regional contractile reserve. GCW, global constructive work; GLS, global longitudinal strain; GWE, global work efficiency; GWI, global work index; GWW, global wasted work; LBBB, left bundle branch block; MW, myocardial work.

LBBB creates a highly heterogeneous regional work pattern: septal segments show reduced constructive work and increased wasted work, reflecting the early, dyssynchronous contraction pattern. Lateral wall segments perform excessive constructive work, compensating for diminished septal contribution but also contributing to elevated GWW due to timing mismatch. This redistribution is one of the hallmarks of LBBB and underlies the rationale for CRT, it aims to restore electrical synchrony, reduce wasted work, and improve global efficiency. Figure 4 presents typical MW parameters in LBBB.

### Clinical implications for CRT selection

The characteristic alterations of myocardial work in LBBB carry important diagnostic and prognostic value in evaluating candidates for CRT. Because MW incorporates both strain and estimated LV pressure, it provides a load-adjusted assessment of mechanical efficiency, improving the detection of true electromechanical dyssynchrony beyond QRS morphology alone. Not all patients with wide QRS complexes exhibit the same degree of mechanical inefficiency. Myocardial work parameters, especially increased GWW and reduced GWE, correlate strongly with mechanical dyssynchrony observed in LBBB. In borderline QRS durations (120–149 ms), MW may help differentiate patients with genuine dyssynchrony from those with merely prolonged electrical conduction. Thus, MW can enhance patient selection, particularly in those with “atypical” or less clear-cut LBBB patterns.

### Myocardial work parameters in prediction of reverse remodeling after CRT

The MW parameters described above appear to be very promising in the context of qualifying patients for CRT and predicting a positive response to therapy. Galli et al. (33) investigated whether GCW, derived from pressure–strain analysis, can identify responders to CRT. The study demonstrated that patients who responded to CRT exhibited significantly higher baseline GCW and greater improvement in GCW after implantation compared with non-responders. GCW outperformed conventional echocardiographic markers of dyssynchrony in predicting reverse remodeling. In this study, GCW below 1,057 mmHg% identified 85% of non-responders with a positive predictive value of 88%. These findings suggest that constructive myocardial work can be a valuable parameter for optimizing CRT candidate selection and assessing therapeutic response.

Another MW parameter, GWW, has also been shown to carry important predictive value in CRT. In the study by Vecera et al. (34), GWW, particularly within the interventricular septum, emerged as a strong marker of mechanical inefficiency in patients with LBBB. Responders to CRT demonstrated markedly elevated septal wasted work at baseline, often exceeding 100%, indicating substantial systolic lengthening and energy loss. Following CRT implantation, GWW significantly decreased in responders, both globally and in the septum, approaching values seen in coordinated ventricular contraction. In contrast, non-responders showed no meaningful improvement in wasted work. Importantly, baseline septal GWW, especially when combined with wall motion score index, was one of the strongest predictors of reverse remodeling, achieving an AUC of 0.86 for CRT response prediction. These findings highlight wasted myocardial work as a promising parameter for optimizing patient selection for CRT and understanding the mechanisms underlying therapeutic benefit.

Duchenne et al. (35) examined 130 CRT patients and showed that therapy causes an acute redistribution of myocardial work, characterized by increased septal work and decreased lateral wall work. This immediate change strongly correlated with long-term reduction in LV end-systolic volume, and the magnitude of lateral-to-septal work shift was the best predictor of reverse remodeling after CRT (model R^2^ = 0.414, *P* < 0.0001). Traditional parameters (QRS duration/morphology, LVEF, ischemic etiology) added little predictive value. The study demonstrates that early correction of regional loading imbalance is the key mechanism driving CRT benefit. Patients showing a marked acute redistribution of work achieved significant reverse remodeling, while those without such changes showed minimal improvement. Assessment of regional MW may therefore enhance CRT candidate selection and improve treatment outcomes.

In another large study with using magnetic resonance imaging, the asymmetry of myocardial work between the septum and the lateral wall emerged as a powerful marker of CRT response (36). Patients with a pronounced lateral-to-septal work difference before implantation were far more likely to experience reverse remodeling, whereas those with minimal asymmetry showed limited benefit. This parameter alone predicted CRT response with good accuracy (AUC 0.77), clearly outperforming conventional markers such as QRS duration or morphology.

In the prospective study of 134 CRT candidates with LBBB and reduced LVEF, regional myocardial work indices, specifically lateral wall constructive work (LW CW) and septal wasted work (septal WW), were identified as the strongest predictors of reverse remodeling and clinical outcomes (37). Both parameters outperformed global myocardial work measures and traditional dyssynchrony markers, and their combination (LW CW >878 mmHg% and septal WW >181 mmHg%) achieved the highest accuracy (AUC 0.832) for identifying CRT responders, who showed a 91% response rate. Low LW CW and low septal WW were also associated with increased risk of death or heart-failure hospitalization at two years. These findings highlight that assessing regional myocardial work more effectively captures the electromechanical substrate relevant for CRT benefit than global indices.

In the another large prospective cohort of 249 patients with HFrEF undergoing CRT, preprocedural GWW emerged as a strong predictor of both CRT response and long-term survival (38). Lower GWW values, specifically GWW <200 mmHg%, were associated with significantly higher rates of CRT nonresponse and a doubled risk of all-cause mortality during a median follow-up of 48 months. A threshold of 200 mmHg% identified nonresponders with high specificity (85%) and remained independently predictive after adjustment for established CRT response determinants (adjusted OR 4.03). Similarly, low GWW independently predicted all-cause death (adjusted HR 2.0) and improved prognostic model performance. Overall, the study demonstrates that low preprocedural GWW reflects unfavorable myocardial mechanics and identifies CRT candidates at increased risk for poor clinical outcomes, suggesting that GWW may enhance patient selection and post-implant monitoring strategies.

Recent evidence shows that also right-ventricular myocardial work (RV MW) derived from noninvasive pressure–strain loops provides an integrated and load-adjusted assessment of RV performance in CRT candidates (39). In this prospective cohort of 91 patients, RV MW was quantified by combining RV longitudinal strain from RV-focused apical four-chamber views with noninvasively estimated pulmonary pressures, enabling calculation of RV global work index (RV GWI), constructive work (RV GCW), wasted work (RV GWW), and work efficiency (RV GWE). Conventional RV parameters, including TAPSE, RV fractional area change, RV GLS, and RV free-wall strain, were also systematically assessed. CRT resulted in significant improvements in RV GWI, RV GCW, and RV GWE, while RV GWW remained unchanged. Importantly, RV GWI was the only RV functional parameter independently associated with CRT response, outperforming RV GLS, RV free-wall strain, and even LV MW indices. Adding RV GWI to the baseline clinical model significantly improved reclassification, highlighting its incremental prognostic value. These findings indicate that RV GWI is a robust and sensitive marker of biventricular functional improvement after CRT and may enhance identification of true responders.

To sum up, patients with: high baseline GWW, low GWE or/and GWI, and pronounced septal-lateral work asymmetry tend to experience more substantial reverse remodeling following CRT. These parameters reflect a greater degree of “reversible inefficiency” that CRT is designed to correct. On the contrary, low levels of wasted work and relatively preserved GWE may indicate that the degree of dyssynchrony is insufficient for CRT to yield meaningful benefit. In such cases, underlying myocardial disease (e.g., diffuse fibrosis, ischemic scar) rather than dyssynchrony may be the predominant driver of LV dysfunction. Myocardial work thus provides a physiological explanation for anticipated non-response, helping refine expectations and avoid unnecessary device implantation.

## Discussion

The evidence reviewed above suggests that deformation-based echocardiography, and especially myocardial work analysis, can describe the mechanical substrate of electromechanical dyssynchrony more precisely than LVEF alone. The disappointing results of the PROSPECT (1) and Echo-CRT (2) trials reduced earlier enthusiasm for echocardiographic dyssynchrony assessment. This largely explains why current guidelines still qualify patients for CRT mainly on the basis of QRS duration, QRS morphology and LVEF (3). It is therefore worth asking why modern strain- and work-based indices might succeed where the parameters tested in PROSPECT did not. PROSPECT relied mostly on M-mode– and tissue Doppler imaging (TDI)–based indices. These indices are angle-dependent, sensitive to noise and translational motion, and showed poor reproducibility both between and within observers and across centres. In a multicentre setting these weaknesses proved decisive, and no single conventional index could overcome them. Echo-CRT then showed that echocardiographically guided CRT in patients with a narrow QRS gave no benefit and was linked to higher mortality. This confirmed that mechanical dyssynchrony without a wide QRS does not identify a treatable electrical substrate.

Speckle-tracking strain differs from these earlier velocity-based indices in several important ways. It is largely angle-independent and more reproducible. Unlike velocity measures, which capture only timing, it measures the actual myocardial deformation and, in the case of myocardial work, its energy cost. Modern strain-based methods do not reduce dyssynchrony to a simple time delay between two opposing walls. Instead, they identify the specific mechanical pattern that CRT is meant to correct: the typical LBBB contraction pattern, septal rebound stretch, and the redistribution of constructive and wasted work between regions. This mechanistic specificity, together with the load-adjusted nature of myocardial work, may explain why recent studies show a more consistent association with reverse remodeling, and why these parameters discriminate responders better than the conventional dyssynchrony indices that failed in PROSPECT.

### Conduction system pacing and the role of deformation imaging

A further development that directly affects the future role of deformation imaging is the rapid emergence of conduction system pacing (CSP), in particular left bundle branch area pacing (LBBAP), as a physiological alternative to conventional biventricular pacing (BiVP). By directly engaging the His–Purkinje system, CSP restores faster and more uniform ventricular activation and a narrower paced QRS than BiVP. A growing body of randomised and observational data suggests that this brings a clinical advantage in HFrEF with LBBB. In the LBBP-RESYNC pilot randomised trial, LBBAP-based CRT produced a significantly greater 6-month improvement in LVEF than BiVP (mean difference ≈5.6%) (40). The large multicentre observational I-CLAS study reported greater reverse remodeling and fewer deaths or heart-failure hospitalisations with LBBAP than with BiVP (41). These findings are supported by later meta-analyses (42) and are reflected in the most recent consensus recommendations on the indications for CSP (43).

Importantly, the echocardiographic tools discussed in this review are not made obsolete by this shift, if anything, they become more relevant. Speckle-tracking measures of mechanical dyssynchrony (mechanical dispersion and the standard deviation of time-to-peak strain) and myocardial work can quantify how much resynchronisation is achieved with CSP, both acutely and during follow-up. In the secondary analysis of the LEVEL-AT trial, conduction system pacing produced significant improvements in mechanical dyssynchrony and global strain over time (23). Pre-implant GLS and myocardial work indices may keep prognostic value for later LVEF recovery in both CSP and BiVP, and the reproducibility of strain imaging makes it well suited to bedside assessment of residual dyssynchrony and to guiding atrioventricular and interventricular optimisation. At the same time, LBBAP is not always fully physiological, some interventricular or septal-to-lateral discoordination may persist. Deformation- and work-based indices are therefore also useful for detecting residual mechanical dyssynchrony that might benefit from LBBB-optimised CRT (LOT-CRT) or device reprogramming. Dedicated prospective studies applying strain and myocardial work to patient selection and response assessment specifically in CSP/LBBAP populations are still limited, however, and represent an important direction for future research.

### Limitations and gaps in knowledge

Several limitations of the current evidence should be acknowledged. First, the lack of a uniform, standardised definition of CRT response, with thresholds based variably on LVESV reduction, LVEF increase, clinical improvement or composite endpoints (Table 1), hampers direct comparison between studies and contributes to the marked heterogeneity of reported predictive values. Second, much of the supporting data derives from relatively small, single-centre and frequently retrospective cohorts with limited external validation. Adequately powered, prospective, multicentre, and ideally randomised, trials testing strain- and myocardial-work–based selection against conventional criteria are still lacking. Third, deformation and myocardial work measurements remain subject to vendor- and software-dependent variability, differences in image acquisition and analysis protocols, and a paucity of standardised reference values, all of which impede reproducibility and cross-centre harmonisation. Finally, myocardial work relies on a non-invasively estimated LV pressure curve derived from cuff blood pressure, which may be inaccurate in the presence of significant valvular disease or markedly altered loading conditions. Until these gaps are addressed through prospective validation and methodological standarisation, the parameters discussed in this review should be regarded as complementary and hypothesis-generating rather than as established, guideline-endorsed criteria for CRT qualification.

## Conclusions

Advanced echocardiographic techniques, especially deformation imaging and myocardial work assessment, offer a more comprehensive and physiologically relevant evaluation of ventricular mechanics than conventional parameters. A key advantage of this advanced diagnostic approach, particularly the use of myocardial work indices, is its ability to overcome the limitations of EF and GLS assessment by accounting for loading conditions. By integrating measures of dyssynchrony, myocardial efficiency, and regional contractility, these tools provide added value in identifying patients most likely to benefit from CRT. Existing evidence supports their role as complementary markers that may refine patient selection and reduce the proportion of non-responders. Continued research in this field is essential to identify the optimal parameters and translate these promising technologies into routine clinical practice.
